# Data for quantitative research of mechanical properties of agar media with concentration gradient, and *Arabidopsis* root growth in these media

**DOI:** 10.1016/j.dib.2022.108383

**Published:** 2022-06-15

**Authors:** Yong Zhou, Meifeng Chi, Haoyang Xiong, Jie Yan

**Affiliations:** Chongqing Normal University, Chongqing 401331, China

**Keywords:** Mechanical properties, Agar media, Stiffness, Resistance, Root growth

## Abstract

The mechanical properties of the plant culture medium affect plant growth and development significantly. The paper presents the data created for the published article entitled “Resistance from agar medium impacts the helical growth of *Arabidopsis* primary roots”. The data contains the real-time output forces of 0.5‒1.2% agar media from Bluehill software, and the forces on the agar surfaces changing with the increase of displacement. Oscillatory rheological experiments were employed to verify the stiffness results of 0.5‒1.2% agar media. Helix diameter and length of roots grown in gradient agar media for Col-0 and DR5-GUS *Arabidopsis* are exhibited.

## Specifications Table

**Table:** 

Subject	Physics
Specific subject area	Root biomechanics
Type of data	Tables, figures
How the data were acquired	Mechanical properties of gradient agar media were measured by Electro PULS E1000 (Instron, USA. Load cell: ± 2 kN Dynacell mounted to base) and Rotational rheometer (Haake MARS, Schwerte, Germany); average length and average helix diameter of 7-day-old roots for Col-0 and DR5-GUS *Arabidopsis* in these media were measured by microscope photograph and image J [1].
Data format	Raw, analyzed
Parameters for data collection	Under the conditions of 23 °C and 50% humidity, uniaxial compression experiment was performed on Electron Puls E1000 machine using Console Bulehill 3 software, and began when the load force touch the sample top area and reached 0.001 N. Loading rate was 1 mm/min. Finish the test when cross-head displacement reached 1.5 mm. Under the same condition, rheological properties of agar media were measured by a rotational rheometer, using 20 mm parallel plates at constant frequency (0.1 Hz) and strain (0.2%).
Description of data collection	The relationship between agar stiffness, agar concentration and root elongation were determined.
Data source location	Chongqing, China
Data accessibility	Mendeley data: https://data.mendeley.com/datasets/ktbcwrsh3r/2
Related research article	Y. Jie, W. Bochu, Z. Yong, H. Shilei, Resistance from agar medium impacts the helical growth of *Arabidopsis* primary roots, Journal of the mechanical behavior of biomedical materials 85 (2018) 43–50. doi:10.1016/j.jmbbm.2018.05.018

## Value of the Data

• This data provide basic reference in determining optimal growing circumstances when cultivating *Arabidopsis* or other plants.
• The data presents information for investigating root growth, buckling and interaction with medium resistance [2].
• Researchers will benefit from the data in investigating the effects of agar rheology on growing plants [3].
• The incorporation of agar in growth medium could be designed in quantitative way and the results of root-gel interaction could be analyzed by physical method.
• To understand the influence of agar stiffness on plant growth in depth, the assessment of mechanical properties of agar made publicly available to make improvement for experiments.

## 1 Data Description

**Fig. 1:**
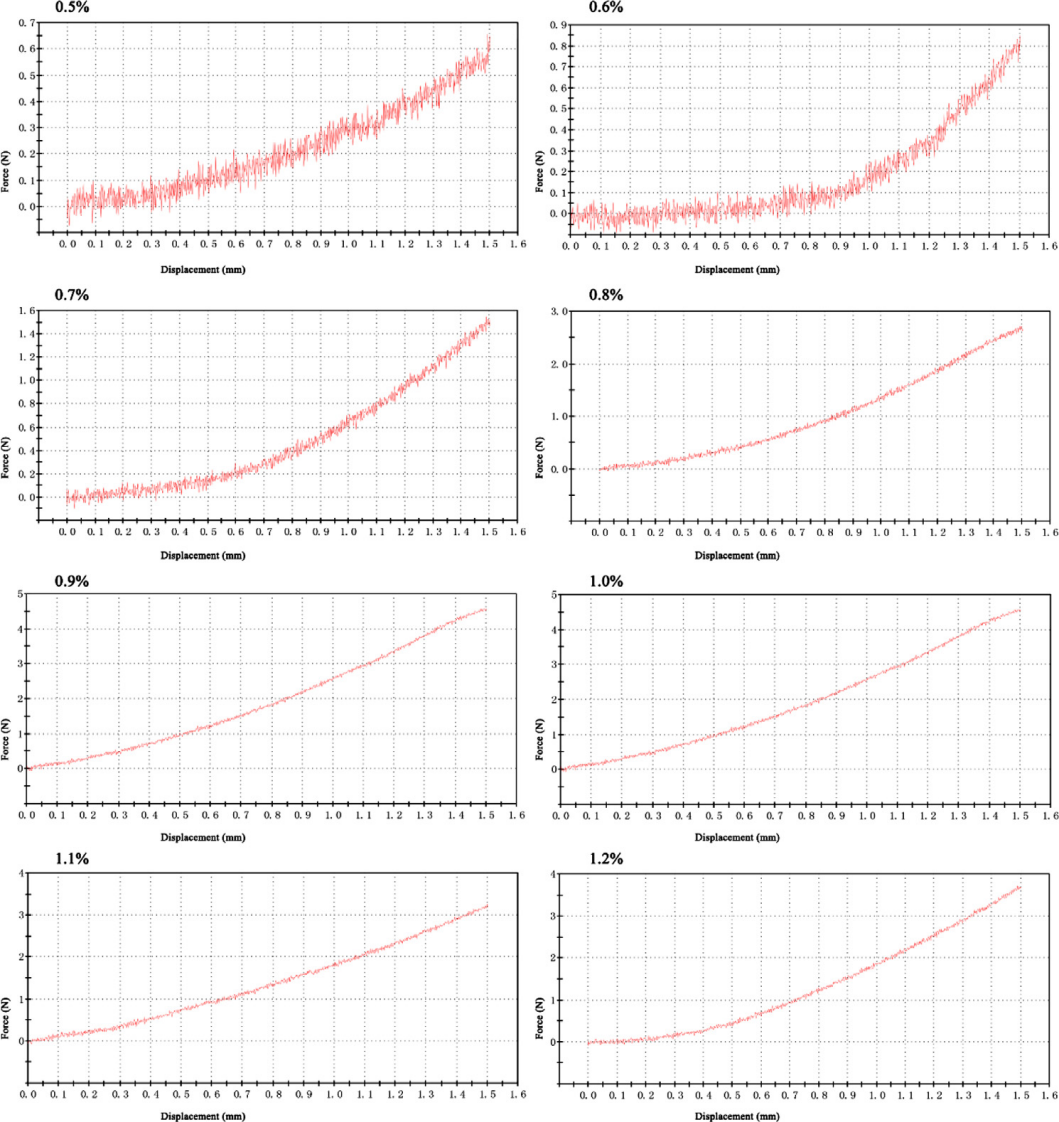
Loading Force-Displacement Curve for 0.5–1.2% agar media from uniaxial compression test. Rectangle samples (length = 40 mm, width = 20 mm, height = 12 mm), area = 800 mm^2^. Common standard tests were used to determine the deformational displacement and Young's modulus. The experiments were repeated more than three times for each agar concentration and similar results to those shown were observed.

**Fig. 2:**
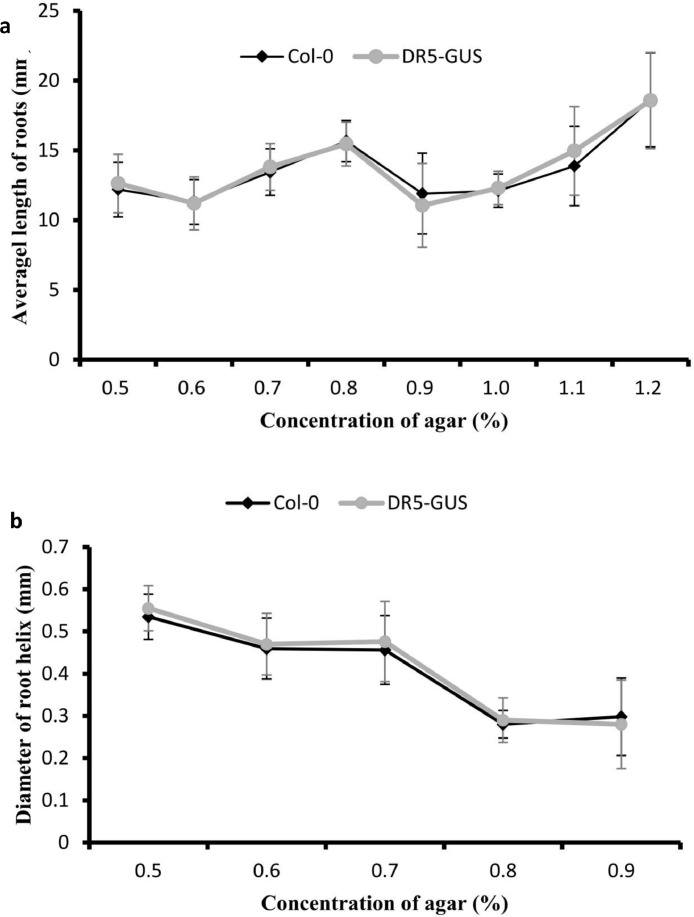
Average length and average helix diameter of 7-day-old primary roots of Col-0 and DR5-GUS in agar media. **a.** Col-0 and DR5-GUS comparison in 0.5–1.2% agar media. **b.** Col-0 and DR5-GUS comparison in 0.5–0.9% agar media. Col-0 and DR5-GUS comparison exhibited no significant difference (t-test, *P* > 0.05).

The data reported herein contains Loading Force-Displacement Curve for 0.5‒1.2% agar media from uniaxial compression test (Fig. 1) and the relationship between agar concentration and moduli (Complex modulus and Elastica modulus) (Table 1). Data of root length (Table 2) and diameter of root helix (Table 3) in each agar media were presented when Col-0 and DR5-GUS *Arabidopsis* grow in agar media on the 7th day. Among the range from 0.5% to 1.2% agar media, no significant difference was found in both dimensions of the helical root and root length between Ecotype Columbia (Col-0) and DR5-GUS seeds (t-test, *P* > 0.05) (Fig. 2). The raw data associated with Fig. 2 were exhibited in Table 2 and Table 3.

**Table 1:** The relationship between agar concentration and moduli (Complex modulus and Elastica modulus). Oscillatory rheological experiments for the effect of agar on the medium rheology were at constant frequency (0.1 Hz) and strain (0.2%).

Concentration of agar (%)	Temperature (°C)	Frequency	Complex Modulus (Pa)			Elastic Modulus (Pa)		
0.5	23	0.5	15025	15166	14988	15000	15169	14698
0.6	23	0.5	17748	17257	17986	17724	17700	17710
0.7	23	0.5	22300	22389	22131	22300	22136	22314
0.8	23	0.5	21824	20997	22011	21824	21820	21832
0.9	23	0.5	25101	25143	25079	24971	24975	24853
1.0	23	0.5	27209	27412	27016	27189	27181	2794
1.1	23	0.5	39867	40054	38465	39766	39754	39789
1.2	23	0.5	39188	39132	39284	39098	39102	39078

**Table 2:** Testing dataset with two genetypes of *Arabidopsis* roots. Length of 7-day-old primary roots of Col-0 and DR5-GUS in agar media with concentration of 0.5–1.2%. This dataset is connected with Fig. 2. a. Col-0 and DR5-GUS comparison in 0.5–1.2% agar media.

	Concentration of agar (%)							
	0.5	0.6	0.7	0.8	0.9	1.0	1.1	1.2
**Root length in *Col-0* (mm)**	14.322	12.297	11.234	14.304	10.802	13.484	13.103	16.764
	9.962	11.333	14.709	13.861	11.311	12.542	12.192	14.879
	10.267	12.53	13.679	17.18	10.426	12.961	11.263	17.738
	13.267	8.307	15.276	16.487	17.754	10.173	11.864	17.507
	10.96	10.926	12.294	14.288	10.201	12.192	16.724	24.478
	10.18	12.21	14.759	14.861	11.786	12.612	13.103	16.764
	11.16	12.29	13.679	17.022	11.121	10.173	12.192	14.879
	10.278	11.333	15.216	14.121	10.456	12.255	11.263	17.738
	14.212	12.53	11.294	15.304	12.213	13.179	11.864	18.507
	10.119	10.307	11.294	15.861	10.802	13.525	13.724	18.278
	10.28	10.93	12.287	17.18	11.311	10.173	12.119	19.049
	13.467	10.322	13.689	16.887	10.426	13.879	13.103	17.738
	9.96	11.451	12.255	14.288	12.754	12.525	12.192	17.507
	10.57	8.406	13.879	14.861	10.201	10.173	11.263	18.278
	13.127	12.296	12.525	16.217	10.936	12.349	11.864	20.325
	14.12	12.429	11.234	16.304	11.567	12.541	13.103	19.738
	13.109	11.433	14.709	13.861	10.802	10.173	12.192	17.507
	14.125	12.223	13.679	17.18	11.311	13.879	11.263	18.258
	11.221	8.707	15.171	16.487	13.426	12.525	11.864	24.121
	13.19	11.926	13.445	14.611	16.754	12.173	12.724	16.241
	12.17	11.035	11.834	13.861	10.601	11.795	12.992	16.984
	13.19	11.219	14.709	15.6604	11.905	12.112	13.875	18.615
	10.113	12.011	13.679	14.318	11.802	13.879	12.119	18.278
	12.712	12.197	15.176	15.821	11.511	12.525	13.103	20.325
	13.129	11.353	11.334	15.324	12.426	10.173	12.192	17.738
	14.222	12.53	14.609	13.927	11.511	10.173	11.263	17.507
	13.946	11.107	13.679	14.288	10.426	11.595	11.864	18.178
	13.497	10.926	15.078	13.861	16.798	12.231	-	24.121
	12.866	11.211	13.453	13.866	11.811	13.171	-	18.258

**Root length in *DR5-GUS* (mm)**	13.978	12.297	12.134	16.487	12.754	10.173	11.864	16.464
	10.196	11.333	14.718	15.611	10.201	11.795	12.724	14.979
	10.267	12.53	13.679	13.861	10.936	12.161	12.992	17.738
	13.267	8.307	15.276	15.6604	11.567	13.484	13.8775	17.507
	10.16	10.926	11.294	14.318	10.802	12.542	12.119	18.278
	10.18	12.21	14.759	15.821	11.311	12.961	13.103	24.121
	11.916	12.29	13.679	15.324	13.426	10.173	12.192	16.241
	10.278	11.333	15.216	13.927	16.754	12.192	11.263	16.984
	14.122	12.151	11.994	14.288	10.601	12.612	11.864	18.615
	12.019	10.307	11.494	13.861	11.905	10.573	13.103	18.278
	10.28	10.913	12.287	14.404	11.802	12.255	12.192	20.325
	13.467	10.322	13.689	13.861	11.511	13.179	11.263	17.738
	9.967	11.451	12.255	16.718	12.426	13.525	11.864	17.507
	10.57	8.406	14.879	16.487	11.511	11.173	16.724	18.278
	13.127	12.096	12.525	14.288	10.426	13.879	13.103	24.121
	14.122	12.429	11.234	15.861	16.798	12.525	12.192	24.478
	13.109	11.433	12.749	17.122	10.802	10.273	11.263	16.764
	14.412	12.223	13.879	14.621	11.311	12.349	11.864	14.879
	11.111	8.907	15.171	15.304	10.426	12.541	13.724	17.738
	12.109	11.926	13.445	14.861	17.754	10.173	12.119	18.507
	12.017	11.435	11.834	17.184	10.201	13.879	13.103	18.278
	13.19	11.219	14.709	16.887	11.786	12.525	12.192	19.049
	11.113	12.211	13.679	14.288	11.121	12.273	11.263	17.738
	12.712	12.197	15.176	14.861	10.456	10.795	11.864	17.507
	13.19	11.353	13.334	16.217	12.213	12.218	13.103	18.278
	14.222	12.573	14.609	16.604	10.802	13.879	12.192	20.325
	13.946	11.107	11.679	14.861	11.311	12.525	11.263	19.738
	12.497	10.926	15.078	17.178	10.426	10.173	11.571	17.507
	12.189	11.305	13.444	15.664	11.905	12.121	13.878	18.615

**Table 3:** Testing dataset with two genetypes of *Arabidopsis* roots with helical growth pattern. Helix diameter of 7-day-old primary roots of Col-0 and DR5-GUS in agar media with concentration of 0.5–0.9%.

	Concentration of agar (%)				
	0.5	0.6	0.7	0.8	0.9
**Diameter of root helix in *Col-0* (mm)**	0.565	0.477	0.417	0.269	0.385
	0.465	0.438	0.312	0.277	0.28
	0.472	0.544	0.469	0.249	0.275
	0.516	0.4	0.573	0.326	0.165
	0.612	0.502	0.573	0.269	0.385
	0.548	0.635	0.472	0.277	0.38
	0.565	0.482	0.417	0.249	0.291
	0.563	0.397	0.435	0.326	0.275
	0.469	0.453	0.424	0.326	0.165
	0.611	0.502	0.521	0.326	0.385
	0.469	0.404	0.415	0.269	0.385
	0.515	0.515	0.415	0.272	0.28
	0.513	0.452	0.427	0.249	0.275
	0.518	0.343	0.443	0.226	0.165
	0.516	0.441	0.417	0.269	0.385
	0.612	0.414	0.431	0.275	0.38
	0.548	0.398	0.415	0.229	0.291
	0.565	0.462	0.412	0.326	0.275
	0.515	0.441	0.417	0.315	0.165
	0.513	0.446	0.437	0.281	0.385

**Diameter of root helix in *DR5-GUS* (mm)**	0.565	0.477	0.417	0.269	0.315
	0.469	0.438	0.312	0.277	0.28
	0.472	0.544	0.469	0.249	0.275
	0.556	0.523	0.573	0.326	0.165
	0.612	0.502	0.573	0.269	0.324
	0.548	0.635	0.472	0.357	0.385
	0.565	0.482	0.417	0.249	0.253
	0.469	0.397	0.477	0.326	0.271
	0.611	0.453	0.417	0.269	0.165
	0.469	0.502	0.332	0.277	0.367
	0.515	0.404	0.569	0.249	0.38
	0.523	0.59	0.553	0.334	0.271
	0.518	0.452	0.573	0.322	0.265
	0.546	0.343	0.472	0.306	0.175
	0.612	0.441	0.412	0.269	0.385
	0.551	0.454	0.435	0.272	0.375
	0.565	0.398	0.424	0.349	0.28
	0.615	0.395	0.551	0.326	0.225
	0.558	0.441	0.515	0.269	0.165
	0.578	0.502	0.415	0.275	0.294

## 2 Experimental Design, Materials and Methods

### 2.1 Agar Medium Preparation

The plant growth medium consisted of 0.5 × Murashige, Skoog basal salts with Gamborg's B5 vitamins (Sigma M-0404), 1.5% sucrose and 0.5‒1.2% agar, and then was adjusted to pH 5.8 with KOH. The agar media were cast into rectangles (length = 40 mm, width = 20 mm, height = 12 mm) for compression test.

Homogeneous agar media with a series of concentration from 0.5% to 1.2% were poured into the petri dishes (Diameter = 9 cm, glass) with thickness of 2 mm [4]. A puncher with 2 cm diameter was pushed vertically into the agar layer to make round agar for oscillatory test [3].

### 2.2 Uniaxial Compression Test and Oscillatory Test

Under the conditions of 23 °C and 50% humidity, axial compression tests were performed on Electro PULS E1000. Loading rate was set as 1 mm/min, and the test was finished when cross-head displacement reached 1.5 mm. The rheological properties were measured using a rotational rheometer [3,5].

### 2.3 Root Length and Helical Root Shape for Col-0 and DR5-GUS Comparison

*Arabidopsis thaliana* roots were growing in agar media with increasing concentration as indicated in reference [1]. Images of roots (n = 30) were captured in every culture condition. Thirty roots from each agar media were tested for root length and diameter of helix. For clear visualization of the root growth pattern in agar media, 7-day-old roots were observed under an Olympus microscope. Measurements of microscopy pictures were conducted by Image J software.

## Ethics Statement

This work didn't involves the use of human subjects. The manuscript adheres to Ethics in publishing standard.

## CRediT authorship contribution statement

**Yong Zhou:** Funding acquisition, Investigation, Methodology, Writing – original draft. **Meifeng Chi:** Resources, Software, Supervision, Validation, Writing – review & editing. **Haoyang Xiong:** Resources, Software, Validation. **Jie Yan:** Conceptualization, Data curation, Formal analysis, Writing – review & editing.

## Declaration of Competing Interest

The authors declare that they have no known competing financial interests or personal relationships which have, or could be perceived to have, influenced the work reported in this article.

## Data Availability

Data for quantitative research of mechanical properties of agar media with concentration gradient, and *Arabidopsis* root growth in these media (Original data) (Mendeley Data).
